# Previously defined variants of uncertain significance may play an important role in epilepsy and interactions between certain variants may become pathogenic

**DOI:** 10.1002/epi4.13085

**Published:** 2024-11-07

**Authors:** Yara Hussein, Hila Weisblum‐Neuman, Bruria Ben Zeev, Shani Stern

**Affiliations:** ^1^ Sagol Department of Neurobiology, Faculty of Natural Sciences University of Haifa Haifa Israel; ^2^ Pediatric Neurology Unit, The Edmond and Lily Safra Children's Hospital Sheba Medical Center Ramat Gan Israel; ^3^ Faculty of Medicine Tel Aviv University Tel Aviv Israel

**Keywords:** epilepsy, next‐generation sequencing, RANBP2, RYR3, SCN9A, variant of uncertain significance

## Abstract

**Objective:**

Epilepsy is a chronic neurological disorder related to various etiologies, and the prevalence of active epilepsy is estimated to be between 4 and 10 per 1000 individuals having a significant role in genetic mutations. Next‐Generation Sequencing (NGS) panels are utilized for genetic testing, but a substantial proportion of the results remain uncertain and are not considered directly causative of epilepsy. This study aimed to reevaluate pediatric patients diagnosed with epilepsy who underwent genetic investigation using NGS panels, focusing on inconclusive variant findings or multiple variants of uncertain significance (VUSs).

**Methods:**

A subgroup of pediatric patients aged 0–25 years, diagnosed with epilepsy, who underwent genetic investigation with an NGS epilepsy panel at the Child Neurology Unit, The Edmond and Lily Safra Children's Hospital, Sheba Medical Center, between 2018 and 2022 through Invitae, was reevaluated. Patients with inconclusive variant findings or multiple VUSs in their test results were included. Genetic data were analyzed to identify potentially pathogenic variants and frequent genetic combinations.

**Results:**

Two unrelated potentially pathogenic variants were identified in the SCN9A and QARS1 genes. A frequent genetic combination, RANBP2&RYR3, was also observed among other combinations. The RANBP2 gene consistently co‐occurred with RYR3 variants in uncertain results, suggesting potential pathogenicity. Analysis of unaffected parents' data revealed certain combinations inherited from different parents, suggesting specific gene combinations as possible risk factors for the disease.

**Significance:**

This study highlights the importance of reevaluating genetic data from pediatric epilepsy patients with inconclusive variant findings or multiple VUSs. Identification of potentially pathogenic variants and frequent genetic combinations, such as RANBP2&RYR3, could aid in understanding the genetic basis of epilepsy and identifying potential hotspots.

**Plain Language Summary:**

We have performed a retrospective analysis on a subpopulation of pediatric patients diagnosed with epilepsy. We found that specific genetic variants were repeatable, indicating their potential pathogenicity to the disease.

**Key points**

- 60% of pediatric patients undergoing genetic testing receive an uncertain result, emphasizing the complexity of gene interpretation in epilepsy diagnostics.
- Pathogenic variants in genes like SCN1A were common, underlining the importance of targeted gene sequencing.
- Variants in genes like SCN9A and QARS1, currently classified as VUSs, showed consistent presence in epilepsy patients, indicating potential pathogenicity.
- Specific genetic combinations, such as RANBP2&RYR3, were frequently observed among uncertain results, suggesting potential pathogenicity.

## 1 INTRODUCTION

Epilepsy is a chronic neurological ailment distinguished by a persistent susceptibility to generate seizures and incur subsequent neurobiological, cognitive, psychological, and social ramifications arising from recurrent seizure occurrences.^1^, ^2^

According to the World Health Organization (WHO), the prevalence of active epilepsy is estimated to be between 4 and 10 per 1000 individuals in the general population. On a global scale, approximately 5 million people are diagnosed with epilepsy yearly.^3^ The majority of epilepsy is diagnosed during pediatric years in which around 1 in 150 children is diagnosed, with the highest incidence rate occurring in infancy.^4^

Epilepsy can be attributed to various causes, but the genetic component is vital; over 50% of epilepsy cases have a genetic component.^5^, ^6^ The literature has described over 500 genes associated with epilepsy, suggesting their potential contribution to the development of the condition.^7^ Various genes were found to be highly associated with epilepsy, including genes coding for sodium channel subunits, GABA receptor subunits, and others.^8^, ^9^ For instance, variants in the SCN1B gene have been shown to cause severe epilepsy, affecting the function and regulation of sodium channels, leading to unstable neuronal excitability.^10^ In addition, variants in the GAD1 gene, recently associated with a severe form of epileptic encephalopathy due to an impaired GABA synthesis, consequently imbalanced neuronal excitability.^11^

Electrophysiological studies on patient‐derived cortical and hippocampal neurons with mutations associated with epilepsy show a neuronal hyperexcitability pattern^12^, ^13^, ^14^, ^15^, ^16^ and a reduction in GABA‐positive neurons.^12^, ^13^

The diagnosis of epilepsy relies primarily on a detailed patient history and neurological examination, while EEGs and neuroimaging like MRI and CT scans serve as critical adjuncts. EEGs, enhanced by state‐dependent recordings and activation procedures, help identify epileptiform abnormalities, though they may require intracranial monitoring for elusive cases. Additionally, metabolic and genetic evaluations are tailored to the seizure type and suspected syndrome, with an increasing role for comprehensive genetic testing in identifying etiologies of epileptic encephalopathies.^17^

Traditional genetic diagnostic tools, including Whole Exome Sequencing (WES) employ sequencing on the protein‐coding regions of a genome through oligonucleotide probes for targeted enrichment of the exome, capturing all coding regions within the genome for sequencing.^18^ Lately, Next‐Generation Sequencing (NGS) panels are becoming a powerful diagnostic tool used for epilepsy, significantly enhancing the diagnostic process.^19^ This method allows for the high‐throughput analysis of DNA and RNA, generating sequencing data rapidly and cost‐effectively compared to traditional methods. NGS panels involve several steps, including DNA fragmentation, library preparation, sequencing by synthesis, and data analysis, enabling the simultaneous sequencing of multiple DNA fragments.^20^ Based on this method, custom panels, in which disease‐relevant genomic regions are sequenced, were designed to focus on particular areas of the genome that are of interest for specific research questions or clinical applications, balancing specificity with cost‐efficiency, contributing to the early detection of epilepsy‐related conditions, as well as other neurodevelopmental disease.^19^, ^21^, ^22^, ^23^ The yield of positive molecular tests is relatively low; about 15%–25% are categorized as pathogenic or likely pathogenic.^24^, ^25^ On the other hand, most of the variants found in these epilepsy panels are categorized as variants of uncertain significance (VUS),^24^ posing a substantial challenge in contemporary genetic variation screening approaches and genetic counseling.

Our work aims to reevaluate a subgroup of tested individuals with inconclusive variant findings or more than one VUS in their panel results, assuming that several rare variants in genetic hotspots can lead to epilepsy manifestation yet not categorized as pathogenic.

## 2 MATERIALS AND METHODS

A retrospective analysis was performed on pediatric patients at the Child Neurology Unit, The Edmond and Lily Safra Children's Hospital, Sheba Medical Center, along with unaffected biological parents, who were genetically tested using the Invitae epilepsy panel as part of clinical assessments between 2018 and 2022. Formal approval was obtained from the Institutional Review Board (IRB) of Sheba Medical Center, ensuring full compliance with established guidelines and standards for research involving human subjects.

### 2.1 Invitae epilepsy testing

Invitae epilepsy tests utilize a targeted gene panel based on NGS technology in which genomic DNA was extracted from blood or saliva samples and enriched for specific regions of interest using a hybridization‐based protocol, followed by sequencing using Illumina technology. The sequencing depth for all targeted regions was set at least 50 times, and the resulting reads were aligned to the GRCh37 reference sequence. The analysis focused on the coding sequence of the indicated transcripts, including a 10‐base pair flanking intronic sequence and specific genomic regions known to be causative of disease. The aim is to identify single‐nucleotide variants (SNVs), short and long indels, exon‐level deletions/duplications, and rare structural rearrangements that disrupt coding sequences performed by Invitae. The panel targets genes associated with syndromic and nonsyndromic causes of epilepsy, generally 187 genes, as depicted in the latest version of Invitae until 2022 with various add‐ons, providing a comprehensive assessment of the genetic factors underlying the condition.

### 2.2 Variants classification

Observed variants are classified as pathogenic (directly contributing to the development of a disease), likely pathogenic (likelihood of causing a known genetic condition), Benign (not known to cause genetic conditions, but can alter the protein products or change the gene's expression), likely benign (is not expected to lead to a genetic condition, but the scientific evidence is not as strong as for the variants that are classified benign) or VUS which is a variation in DNA that has an uncertain or unknown impact on health as provided by Invitae. These classifications are based on *Sherloc*‐ Invitae's variant classification algorithm, based on the initial American College of Medical Genetics and Genomics/Association for Molecular Pathology (ACMG/AMP) classification framework, and represent the industry standard among clinical genetic testing laboratories^26^ Additionally, in order to assess the potential functional impact of the identified VUS by *Sherloc*, we applied PolyPhen‐2 (Polymorphism Phenotyping v2), which predicts the potential damaging effects of amino acid substitutions on protein structure and function by analyzing evolutionary conservation, sequence features, and structural characteristics of the protein.^27^ To minimize uncertainty in genetic testing, follow‐up testing was done for selected unaffected biological parents of patients previously tested.

### 2.3 Observed variants analysis

The electronic tests of 156 probands and 74 unaffected biological parents were used. Data were extracted using custom‐written MATLAB scripts (R2023a, Mathworks) and anonymously analyzed (included and excluded data are shown in Figure S1a).

Tests were grouped into three groups: Positive tests (in which there was a detection of at least one pathogenic/likely pathogenic variant that directly contributes to the emergence of epilepsy), Negative tests (no detected variant in the selected genes or only benign or likely benign detected variants) and Uncertain tests (at least one detected VUS). For each, the total number of detected variants was counted, and the most definitive leaders were found and compared to their population incidence based on the Genome Aggregation Database (gnomAD) browser (https://gnomad.broadinstitute.org/) and the Exome Aggregation Consortium (ExAc) database. Variants with allele frequency <0.1% were considered rare. Protein network path analysis was performed to find significant pathways against the Kyoto Encyclopedia of Genes and Genomes (KEGG) and Gene Ontology (GO) databases for observed pathogenic and uncertain variants separately. The Network Analyst web application was used to create a graphical representation of Protein network path analysis (https://www.networkanalyst.ca).

In the collective findings, the most prevalent combinations of VUS were identified across each subgroup. An identical analysis was conducted on probands who possessed data for both unaffected biological parents, aiming to identify notable combinations of genetic variants; genetic correlations were calculated using Correlation AnalyzeR (https://gccri.bishop‐lab.uthscsa.edu/shiny/correlation‐analyzer/).

## 3 RESULTS

One hundred fifty‐six pediatric patients at the Child Neurology Unit of The Edmond and Lily Safra Children's Hospital, Sheba Medical Center, underwent genetic testing using the Invitae epilepsy panel, with 94% of the patients providing blood samples. Approximately half of the patients were female (53.2%), and the average age at the time of testing was 7.6 years with a standard deviation of 5.3 years (range: 0–25 years). The epilepsy panel, which typically includes 187 genes, was sometimes supplemented with additional panels or add‐ons, resulting in an average of 227.4 genes being tested. The genetic panel tests were conducted for various indications, including refractory epilepsies, infantile spasms, and self‐limited childhood focal epilepsies accompanied by electrical status epilepticus in sleep (ESES), as well as focal and generalized epilepsies. Most patients had refractory epilepsy, while a minority responded to monotherapy. Additionally, the patients exhibited a diverse range of other neurological manifestations, including developmental delay, autistic spectrum disorder (ASD), intellectual disability, mood disorder, anxiety, learning difficulties, speech difficulties, behavioral difficulties, attention deficit and hyperactivity disorder (ADHD), ticks, headaches, weakness episodes, dizziness, and tinnitus.

**FIGURE 1:**
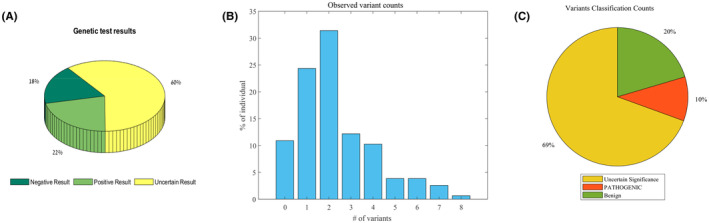
A graphical description of the probands data. (A) Test outcomes categorized according to the observed genetic variants. (B) Observed genetic variants in the targeted genes on the NGS INVITAE panel ranged from zero to 8 per individual. (C) A classification of the observed variants.

A total of 18% of the probands who underwent testing received a negative result, indicating no detectable genetic variants related to epilepsy in the performed panel; 22% of the probands obtained a positive outcome, indicating the presence of at least one pathogenic variant associated with epilepsy. The majority, constituting 60% of the probands, received an uncertain result (see methods), as shown in Figure 1A and Figure S1c.

On average, 2.3 + 1.7 genetic variants were observed across the probands. Notably, 11% of the individuals showed no observed variants in the targeted genes, while almost 65% exhibited two or more observed variants, as shown in Figure 1B. Among all the observed variants, 20% were classified as benign or likely benign, 10% were classified as pathogenic or likely pathogenic, and the majority, constituting 69%, were classified as VUSs, as shown in Figure 1C.

### 3.1 Regulation of action potential and multiple neuronal developmental processes are dysregulated in child epilepsy

**FIGURE 2:**
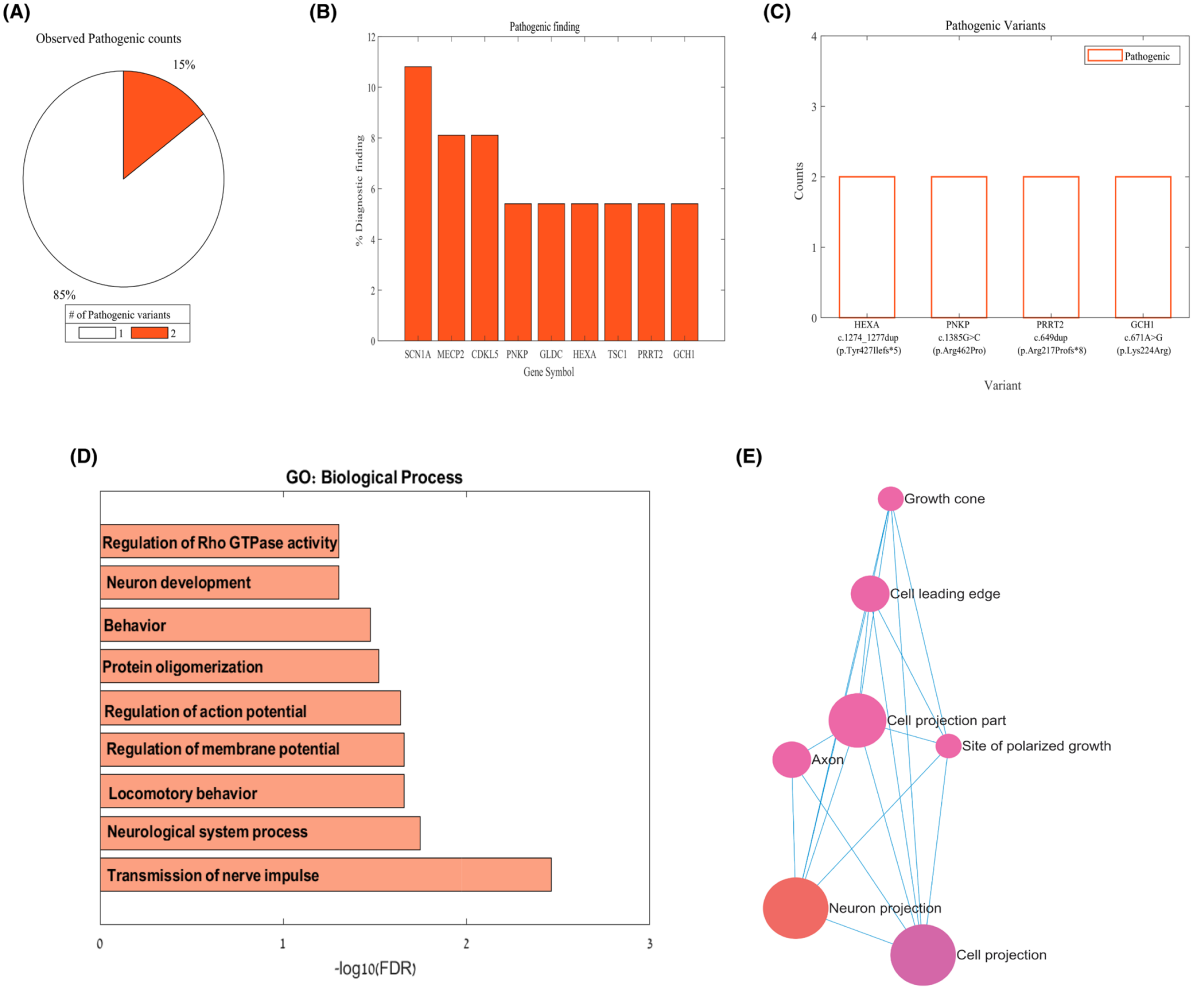
Diagnostic results and genes with pathogenic findings. (A) Among individuals with positive results, the observed pathogenic variants in the targeted genes on the NGS INVITAE panel ranged from 1 pathogenic variant responsible for the condition to two pathogenic variants per individual. (B) The pathogenic genes were repeated more than three times in the positive proband data. (C) The pathogenic variants were repeated more than once in the positive proband data. (D) Significant enrichment of affected GO biological process in the set of pathogenic genes (FDR < 0.05). (E) Significant enrichment of affected GO cellular components in the set of pathogenic genes (FDR < 0.05).

As previously mentioned, 22% of the tests were positive; 15% exhibited the presence of two different pathogenic variants, while 85% indicated the presence of a single pathogenic variant contributing to the onset of epilepsy (Figure 2A). Among the pathogenic genes identified in the probands, the SCN1A gene‐coding for the α‐subunit of a neuronal voltage‐gated sodium channel^28^ was detected in 11% of the positive results, followed by MECP2 and CDKL5 genes, each observed in 8% of the cases, as shown in Figure 2B. Noteworthy the pathogenic HEXA(NM_000520.6): c.1274_1277dup, p.(Tyr427Ilefs*5) variant, the PNKP(NM_007254.4):c.1385G>C, p(.Arg462Pro) variant, the PRRT2(NM_145239.3):c.649dup, p.(Arg217Profs*8) variant, and the GCHI(NM_000161.3):c.671A>G, p.(Lys224Arg) variant were observed twice each in the positive tests (Figure 2C). Notably, mutations in the HEXA and PNKP genes are associated with autosomal recessive conditions. In contrast, mutations in the PRRT2 and GCHI genes are associated with autosomal dominant conditions, suggesting their potential pathogenicity role in explaining the epileptic condition.

Based on the detected pathogenic genes in our tests, Cellular Components (CC) related to neuron projection, cell projection, axon, growth cone, and others showed significant GO: CC enrichment (FDR < 0.05). In terms of biological processes (BP), the transmission of nerve impulses, neurological system processes, regulations for action and membrane potentials, and neuron development were enriched (FDR < 0.05), as shown in Figure 2D,E, indicating several neuronal alterations^29^ contributing to the development of epilepsy.

### 3.2 Two genetic alterations in the SCN9A and QARS1 genes, currently classified as VUS's, have a potential implication for pathogenicity in child epilepsy

**FIGURE 3:**
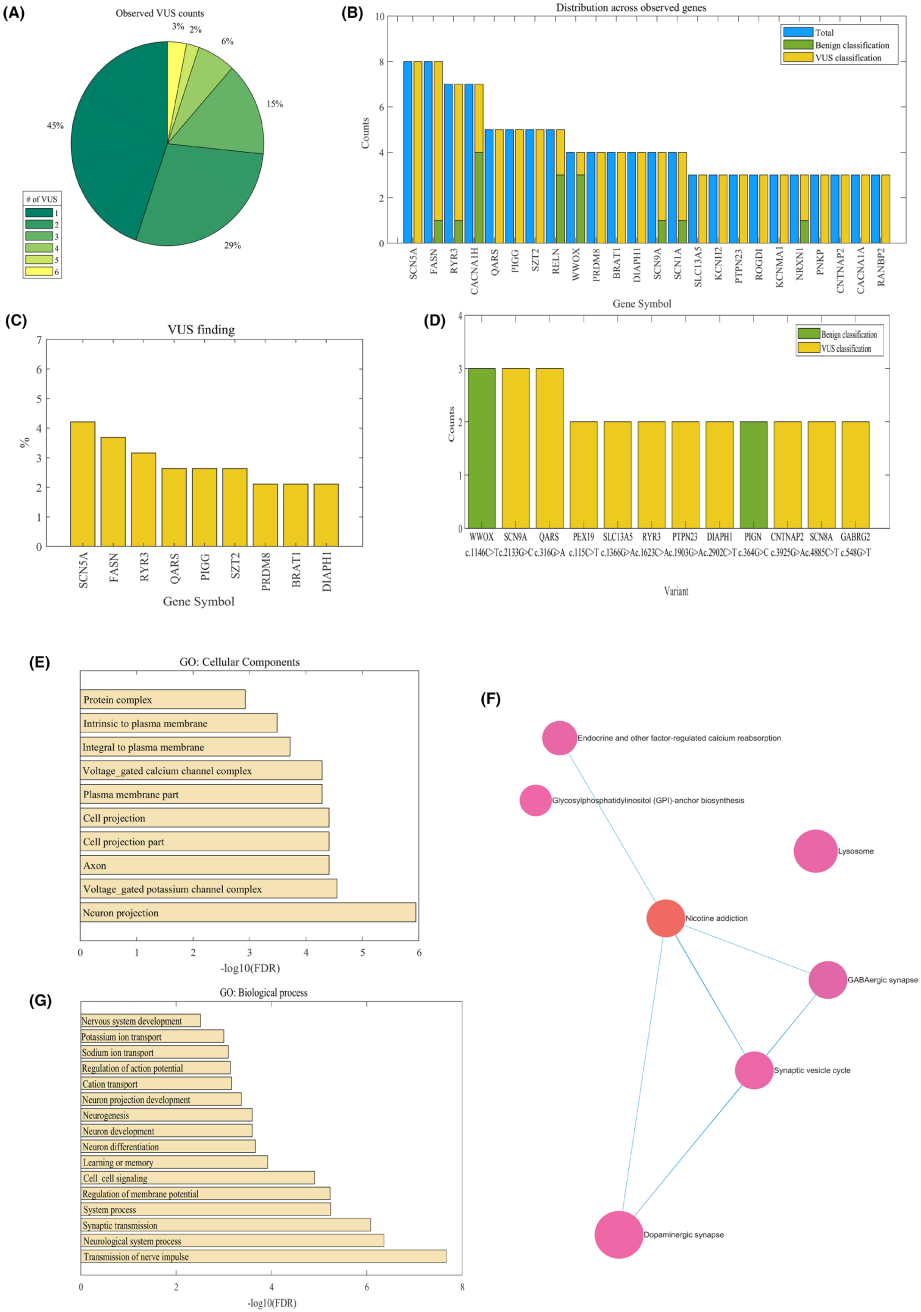
Uncertain results and genes with uncertain findings. (A) Among individuals with uncertain results, the observed uncertain variants in the targeted genes on the NGS INVITAE panel range from one to six variants per individual. (B) The frequent genes in the uncertain data. (C) The frequent genes with uncertain yield. (D) The uncertain Variant yield frequencies. (E) Significant enrichment of affected GO cellular components in the set of VUS genes (FDR < 0.05). (F) Significant enrichment of affected KEGG pathways in the set of pathogenic genes (FDR < 0.05). (G) Significant enrichment of affected GO biological process in the set of VUS genes (FDR < 0.05).

For the uncertain results with at least one detected VUS, an average of 2 ± 1.2 VUSs were present in the patients' panels, out of which 45% showed the presence of a single VUS, while 55% exhibited two or more VUSs, as presented in Figure 3A. SCN5A, FASN, and RYR3 were the most frequently observed VUS genes, as shown in Figure 3B,C. Furthermore, the SCN9A (NM_002977.4):c.2133G>C, p.(Leu711Phe), and the QARS1(NM_005051.3):c.316G>A, p.(Asp106Asn) variants were observed three times each in the uncertain tests, along with other variants shown in Figure 3D. These variants appear to have a high penetrance in epilepsy patients compared to their presence in the general population based on the gnomAD and ExAc databases (*p* = 3.76e‐07), although classified with an uncertain significance. The clinical background of affected patients is shown in Tables S1, S2.

In terms of GO biological processes, the detected VUS showed enrichment in processes related to the transmission of nerve impulses, neurological system processes, regulations for action and membrane potentials, cell signaling, synaptic transmission, neuron development, and differentiation, among others. Furthermore, various neuronal components and channels, such as voltage‐gated calcium and potassium channels, were enriched in terms of GO: CC (FDR < 0.05) (Figure 3E,G). Notably, KEGG pathways associated with different synapses, including GABAergic and dopaminergic synapses and the synaptic vesicle cycle, showed significant enrichment (FDR < 0.05) as shown in Figure 3F.

### 3.3 RANBP2 and RYR3 variant combination as a risk factor for child epilepsy

**FIGURE 4:**
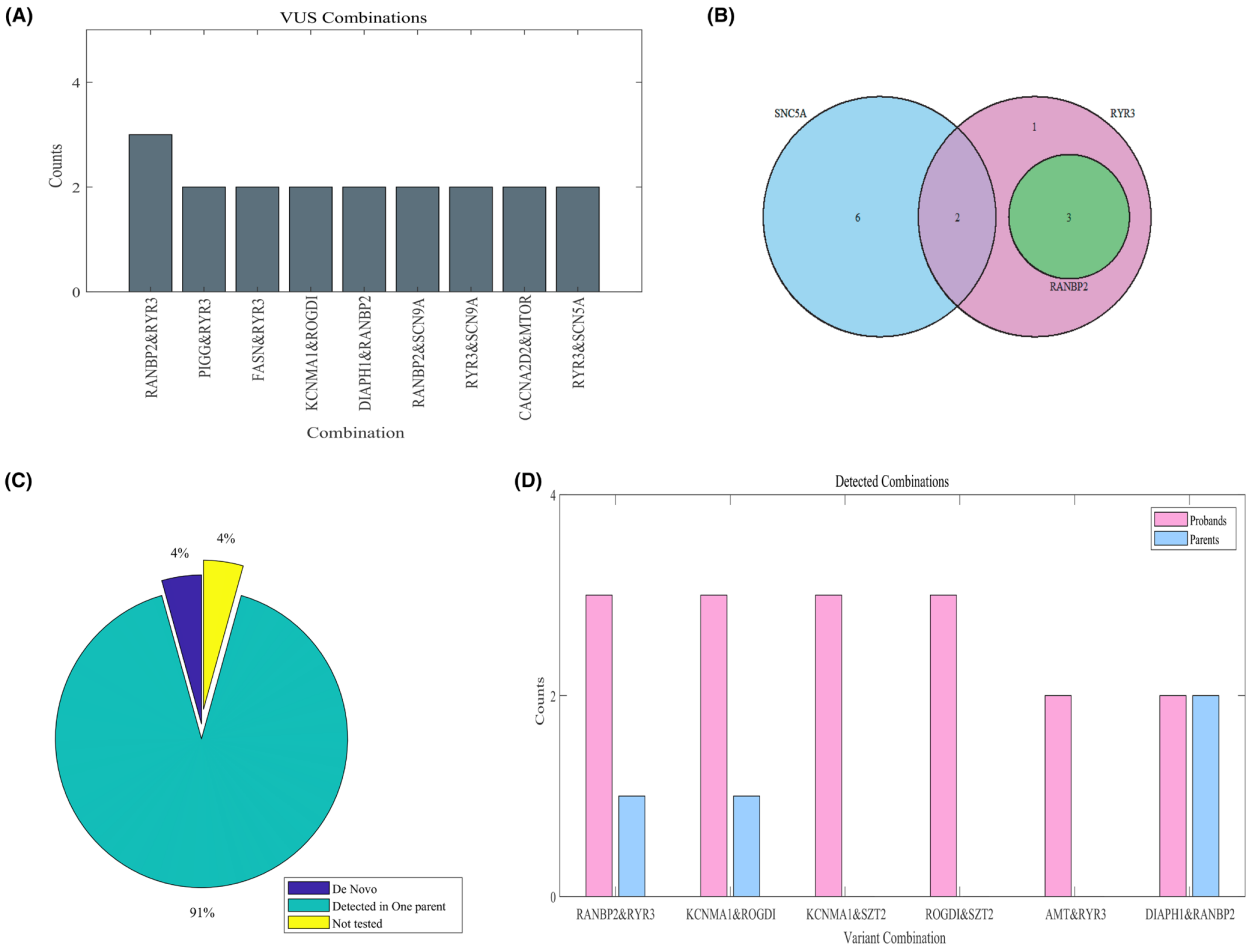
Genetic combinations as a hotspot for child epilepsy. (A) Frequent genetic combinations among affected children. (B) Frequent genetic combinations among uncertain results in the SCN5A, Ryr3, and RANBP2 genes. (C) Distribution of de novo, detected and not tested variants in parent's data. (D) Frequent genetic combinations among individuals and unaffected biological parents.

Upon analyzing the frequent combinations of VUSs in the overall results, it became evident that the combination of the RANBP2 and RYR3 variants occurred most frequently; it was observed in three patients out of 156 in our data. Additional combinations of VUS are shown in Figure 4A. Similarly, the RANBP2 & RYR3 combination remains the most prevalent when focusing solely on uncertain results, as shown in Figure 4B. Interestingly, all detected variants of the RANBP2 gene were designated as VUSs and coexisted in diverse combinations with the RYR3 variants. The clinical background of affected patients is shown in Table S3. Briefly, these patients exhibited a range of neurodevelopmental and neurological phenotypes, including generalized epilepsy, Lennox–Gastaut syndrome, and suspected temporal auditory epilepsy—notably, all patients presented with developmental challenges such as speech, learning, and behavioral difficulties. Two patients were diagnosed with ADHD, while one had suspected autism without a formal diagnosis.

In a subgroup of selected probands (*n* = 38) within the cohort, genetic testing was conducted on both unaffected biological parents (*n* = 74) to examine the variants identified in their children. Surprisingly, 91% of the observed proband variants were also present in one unaffected parent, while 4% were determined to be de novo, indicating that they were not present in either parent, as shown in Figure 4C. To explore potential combinations that might contribute to the risk of developing epilepsy, optional combinations in the parental data were analyzed. The combination of RANBP2 and RYR3 stood out prominently among the observed variants in the proband data, as illustrated in Figure 3D. We noted a single distinct inherited genetic combination, each originating from different parents, suggesting a potential genetic basis for the child's epilepsy. Furthermore, there was a single case where a parent shared the same genetic combination as their affected child despite not having an epilepsy diagnosis. Moreover, other combinations in probands' data exhibited potential risk factors compared to their unaffected biological parents (*p* = 6.7471e‐04).

## 4 DISCUSSION

In the past decade, significant progress has been made in identifying gene mutations associated with epilepsy and unraveling the molecular mechanisms that contribute to the clinical presentation of the disease. It is becoming increasingly evident that comprehending these mechanisms is crucial for selecting optimal treatment approaches for affected individuals. Consequently, genetic testing is done to determine whether these features are associated with a known genetic condition.^30^

Epilepsy is a disorder with a notable genetic component,^6^ has been linked to numerous genes known to play a direct role in its development. NGS panels have been developed to facilitate the identification of potential pathogenic variants in these epilepsy‐associated genes. These panels are designed to probe genes highly relevant to epilepsy, allowing for a targeted and comprehensive analysis of genetic variations^31^ such as Invitae's epilepsy panel.

In this study, we analyzed genetic data from patients diagnosed with infantile and childhood epilepsy who had undergone Invitae's tests. These samples were obtained either from blood or saliva, some with confirmed genetic alterations correlated with epilepsy and some with no definite genetic condition. It is essential to highlight that the tests' results exhibited partial ambiguity, with approximately 60% of the tests (and 69% of the detected variants) showing significant uncertainty. One relatively unique characteristic of VUSs is that while the result itself may remain static, its meaning is often resolved over time as more data are gathered.^30^ This complex task is crucial for comprehending the underlying pathogenic elements, enabling the understanding of involved mechanisms, and implementing the appropriate treatments, which can lead to effectively managing the disorder.

Consistent with previously reported data,^32^ we observed that the SCN1A gene was one of the leaders in the observed pathogenic variants resulting in epilepsy seizures associated with Dravet syndrome (DS)^33^ and other epilepsy syndromes.^34^

Upon analyzing the VUSs reported, we observed that the SCN9A (NM_002977.4):c.2133G>C, p.(Leu711Phe) variant was repeated in 3% of our tests. This sequence change replaces leucine with phenylalanine, which are both neutral and non‐polar amino acids at codon 711 of the SCN9A protein (rs772492538, gnomAD 0.003%, ExAc 0.002% PolyPhen‐2 prediction: Probably Damaging^35^) potentially altering the function of the Nav.17 sodium channel, leading to signal disruptions.^36^ Similarly, a repeated unrelated variant is QARS1(NM_005051.3):c.316G>A, p.(Asp106Asn), inherited in an autosomal recessive pattern and associated with Microcephaly, progressive, seizures and cerebral and cerebellar atrophy syndrome (OMIM #615760).^37^ We also observed that this variant was repeated in 3% of our tests. This sequence change replaces aspartic acid, which is acidic polar, with asparagine, which is a neutral polar amino acid, at codon 106 of the QARS protein (rs141983717, gnomAD 0.02%, ExAc 0.03%). According to ACMG guidelines, although this variant is classified as a VUS, its rare occurrence in population databases does not rule out potential pathogenicity, especially in digenic inheritance or variable phenotypes. The consistent observations of these variants in multiple unrelated patients indicate their potential pathogenicity, leading to their consideration as a causative variant responsible for epilepsy development. It is noteworthy that these specific variants have not been reported as associated with epilepsy yet.

While both variants may not yet be directly linked to epilepsy, both are associated with broader neurological conditions. Given the repeated observations of these variants in unrelated patients, we hypothesize that it may contribute to epilepsy through mechanisms involving multiple genetic loci.

Furthermore, based on the set of detected VUSs, dysregulation of several neurodevelopmental functions and GABAergic synapse enrichment were observed, as previously reported.^34^ A decreased activity of the inhibitory circuitry, mainly GABAergic, is thus likely to be a significant factor contributing to seizure generation in affected patients.^33^

Nearly half of the patients with an uncertain result showed the presence of two or more VUSs among their detected variants, highlighting the importance of precise interpretation regarding the clinical relevance of various variant combinations. Notably, the combination of RYR3 and RANBP2 variants was observed multiple times in our data, standing out among all possible combinations. Interestingly, the RANBP2 variants, when present as VUS, consistently co‐occurred with a variant of RYR3, suggesting a potential pathogenic role for this combination in contributing to the manifestation of epilepsy.

The RANBP2 gene (on chromosome 2q11‐13) encodes the nuclear pore component of RAN binding protein 2^38^ and is associated with acute necrotizing encephalopathy, an autosomal dominant ailment characterized by brain damage that usually follows an acute febrile disease.^39^ The RYR3 gene is a ryanodine receptor predominantly expressed in the brain and functions as a critical regulator of calcium release from intracellular reservoirs. Its involvement in synaptic plasticity is well‐established, and studies with RYR3 knockout mice have demonstrated compromised spatial learning abilities.^40^

Following analyzing the detected variants from unaffected biological parents' tests, it was observed that one RYR3 & Ranbp2 combination was inherited, with each variant originating from a different parent, suggesting that the combination of genes may be the cause of the child's epilepsy. Notably, there was a single instance where one parent possessed the same combination as their affected child despite not having a diagnosis. The increased prevalence of this genetic combination, relative to other potential combinations, suggests its potential role as a trigger or risk factor for disease development. However, it is crucial to note that the genetic correlation between these genes is relatively low (Correlation AnalyzeR, *r* = 0.029; *p* = 0.00805).^41^ Given the absence of physical interaction between RYR3 and RANBP2, we hypothesize that the co‐occurrence of these variants may result in a downstream effect mediated by a third gene or pathway. RYR3, known for its role in calcium signaling and synaptic plasticity, and RANBP2, associated with nuclear transport and encephalopathy, may converge in their impact on neural function via shared pathways, such as calcium homeostasis, neuronal signaling, or stress response pathways.

Despite this, the observed pattern raises the possibility that specific gene combinations may influence disease manifestation or susceptibility. Further investigations are required to explore potential mediators of this gene combination, possibly involving whole‐exome sequencing or multi‐omics approaches to uncover additional genetic or molecular factors contributing to the observed phenotypes.

## 5 CONCLUSION

Identifying potentially pathogenic variants like those found in SCN9A and QARS1, as well as frequently co‐occurring combinations like RYR3 and RANBP2, offers valuable insights into the genetic foundations of epilepsy. This approach presents a pivotal tool for uncovering new genetic risk factors and refining diagnostic precision as our comprehension of genetic interactions evolves. Future studies should authenticate these findings in larger cohorts and explore integrating reanalysis into routine clinical practice to enhance diagnostic outcomes for undiagnosed epileptic patients.

### 5.1 Limitations

Our analysis included patients' data assessed by INVITAE epilepsy panels, which are specific for targeting potential epilepsy‐related variants. Additionally, we had a limited cohort due to the end of contracts with INVITAE.

### 5.2 Clinical relevance or future directions

Our work contributes to understanding the genetic basis of epilepsy, emphasizing the importance of genetic testing and targeted analysis through NGS panels. Identifying specific pathogenic variants in genes such as SCN1A, SCN9A, and QARS1 and the recurrent combination of RANBP2 and RYR3 variants underscores the necessity for ongoing research to elucidate their roles in epilepsy development. Suggesting that comprehending genetic interactions and their implications could lead to improved diagnostic strategies, personalized treatment approaches, and more effective management of epilepsy.

## AUTHOR CONTRIBUTIONS

SS designed, directed the study, and edited the manuscript; YH analyzed the data and wrote the manuscript; BBZ and HWN provided the electronic INVITAE tests and reviewed the manuscript.

## FUNDING INFORMATION

Zuckerman STEM Leadership Program and Israel Science Foundation (ISF) grants 1994/21 and 352/21 for Prof. Stern.

## CONFLICT OF INTEREST STATEMENT

None of the authors has any conflict of interest to disclose. We confirm that we have read the Journal's position on issues involved in ethical publication and affirm that this report is consistent with those guidelines.

## ETHICS STATEMENT

This study was approved by the IRB of Sheba Medical Center, which ensures full compliance with established guidelines and standards for research involving human subjects due to the retrospective approach.

## Supporting information


Figure S1.

Table S1.

## Data Availability

Data supporting this study's findings may be available to the corresponding author upon reasonable request.
