# Supplementary figures and images for: Previously defined variants of uncertain significance may play an important role in epilepsy and interactions between certain variants may become pathogenic
Source: Epilepsia Open. 2024 Nov 7;9(6):2443–53. doi: 10.1002/epi4.13085 (PMC11633689; doi:10.1002/epi4.13085)

a

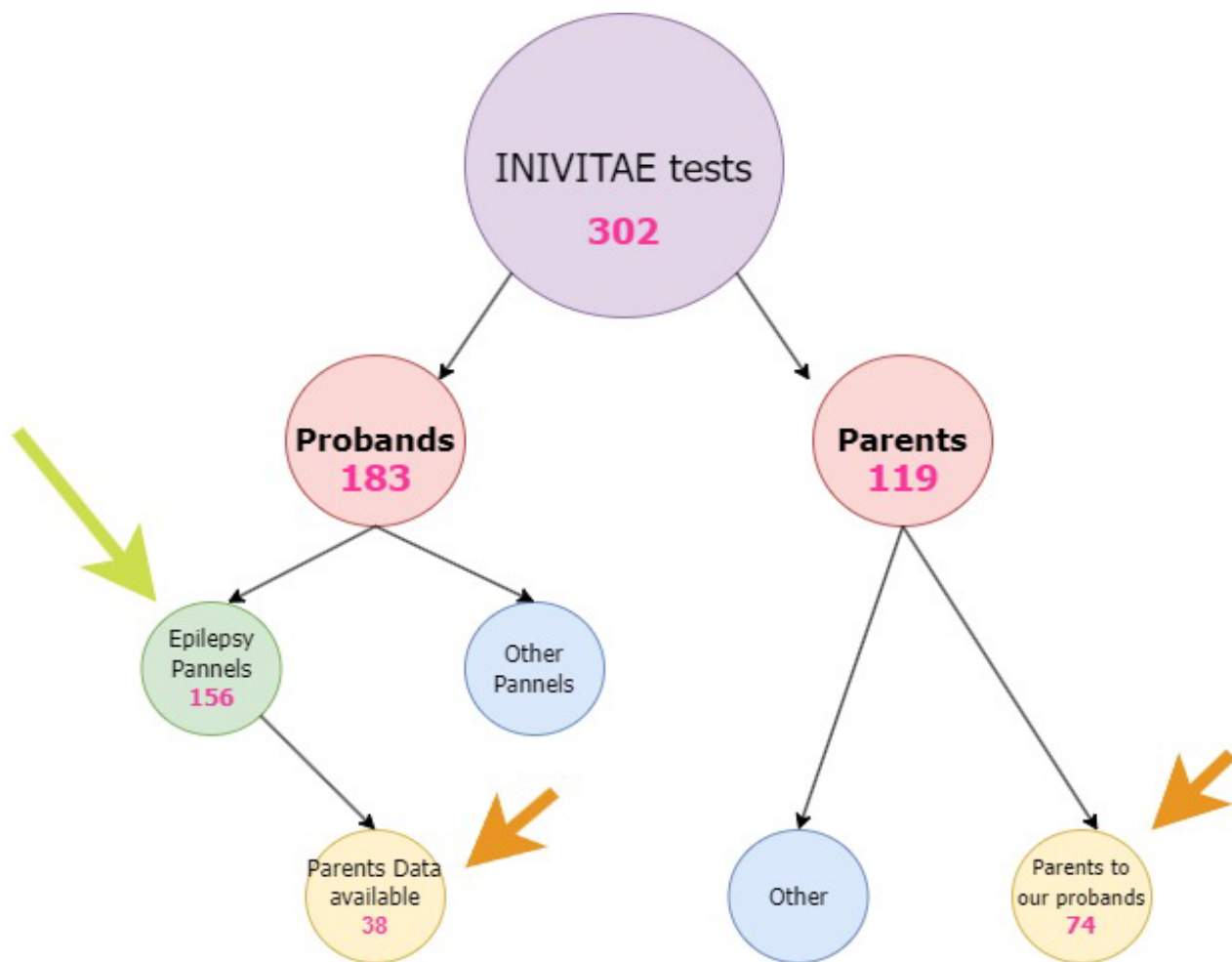

b

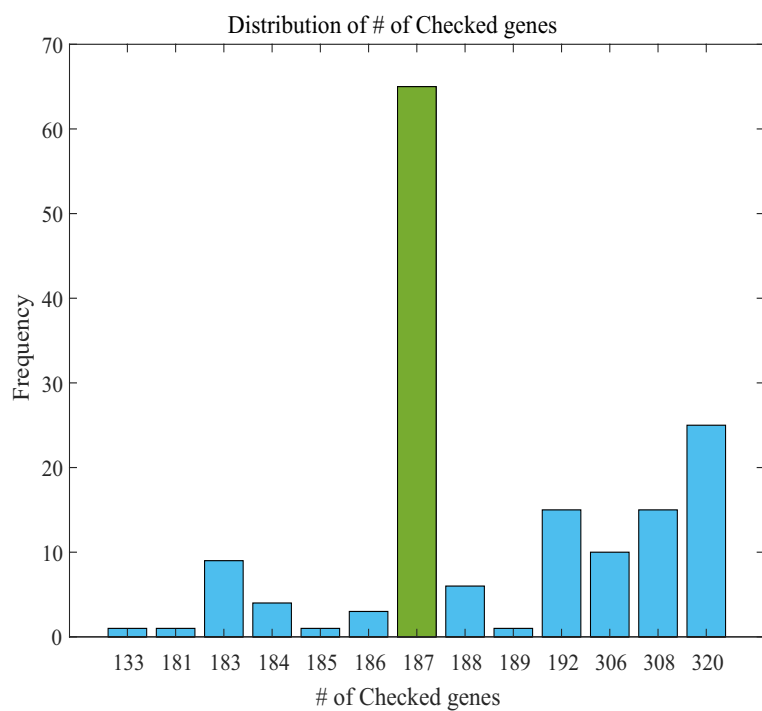

c

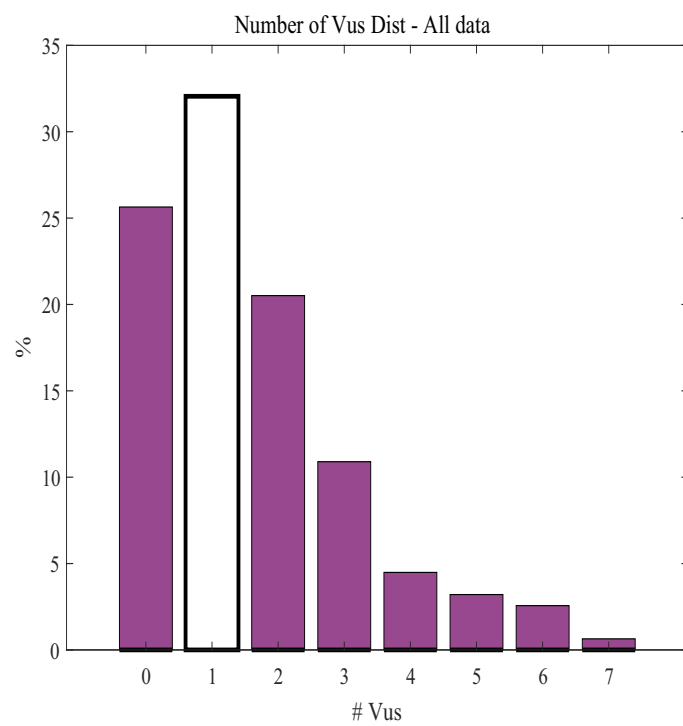

Supplement: Supplementary file 1 — Figure S1. [file EPI4-9-2443-s001.pdf]
