# Supplementary material for: Previously defined variants of uncertain significance may play an important role in epilepsy and interactions between certain variants may become pathogenic
Source: Epilepsia Open. 2024 Nov 7;9(6):2443–53. doi: 10.1002/epi4.13085 (PMC11633689; doi:10.1002/epi4.13085)
Supplement: Supplementary file 2 — Table S1. [file EPI4-9-2443-s002.docx]

Supplementary Table 1.

c.2133G>C in SCN9A

|  | Clinical information | Genetic variants |
| --- | --- | --- |
| Case 1 | Manifested with Rolandic epilepsy and ESES. He had learning, speech, behavioral difficulties, and suicidal ideation. MRI was normal. He had refractory epilepsy to multiple medications, with regimen of Levetiracetam, Clobazam and Diazepam that controlled his seizures | Additional two VUS were identified: c.2339T>C (p.Leu780Pro) in GRIN2A and c.7939G>A (p.Gly2647Ser) in RELN genes; all mentioned VUSs were inherited from the father |
| Case 2 | Manifested with multiple tonic and staring seizures. She had microcephaly, Severe intellectual disability, and was diagnosed with autism. Her MRI showed bilateral opercular ischemia. Her epilepsy was not fully controlled but stable with Levetiracetam | An additional four VUS in the epilepsy panel were identified: c.13120_13122del (p.Lys4374del) in RYR3, c.568C>T (p.Leu190Phe) in NGLY1, c.142T>A (p.Phe48Ile) in CNTN2 and c.44G>C (p.Arg15Thr) in SCN9A genes. No parental data is available. |
| Case 3 | Manifested with generalized epilepsy. She had learning, speech, ADHD, and autism. Her epilepsy was well controlled with Valproic acid | The c.2133G>C variant in the SCN9A gene was inherited from the father. An additional three VUS in the epilepsy panel were identified: c.2902C>T (p.Pro968Ser)in KCNH2, c.461A>G (p.Asp154Gly) in PEX16 and c.824C>T (p.Thr275Met) in PRICKLE1 genes, all inherited from the mother. |

Supplementary Table 2.

c.316G>A variant in the QARS1 gene

|  | Clinical information | Genetic variants |
| --- | --- | --- |
| Case 1 | Having this sole variant detected as VUS manifested with Rolandic epilepsy. He had speech and behavioral difficulties, he had anxiety, ADHD, and autism. MRI was normal. He had refractory epilepsy to multiple medications, with a regimen of Sulthiame and Clobazam that controlled his seizures. No parental data is available. | He had 1q24.3 deletion and 9q34.3 duplication in CMA, which doesn't explain his clinical manifestation. |
| Case 2 | Manifested with Infantile spasms. She had a developmental delay. Her MRI was normal. Her hypsarrhythmia was controlled with ACTH | An additional two VUS in the epilepsy panel were identified: c.2902C>T (p.Arg968Cys) in DIAPH1 And c.6035G>A (p.Arg2012His) in SCN5A genes. No parental data is available/ |
| Case 3 | Manifested with focal epilepsy with generalization. He has mild speech delay and juvenile idiopathic arthritis (JIA). His epilepsy was well controlled with Lacosamide. MRI showed Rt cortical dysplasia | An additional VUS in the epilepsy panel was identified:c.115C>T (p.Pro39Ser) in PEX19 gene. No parental data is available |

Supplementary Table 3.

RYR3 & RANBP2

|  | Clinical information | Genetic variants |
| --- | --- | --- |
| Case 1 | c.7513C>A (p.Pro2505Thr) in RANBP2 and c.1235G>A (p.Arg412Gln) in RYR3 genes were detected. Manifested with generalized epilepsy. He had speech, learning, and behavioral difficulties, he had anxiety, ADHD, and suspected autism without a formal diagnosis. He had migraine. He was well controlled with valproic acid and finally withdraw the medication | An additional VUS was identified: c.1862C>T (p.Ala621Val) in the PIGG gene. No parental data is available. |
| Case 2 | c.231C>G (p.Asp77Glu) in RANBP2 and c.3128C>G (p.Thr1043Ser) in RYR3 genes were detected. Manifested with lennox gastaut syndrome. He had speech, learning, and behavioral difficulties, ADHD, and intellectual disability. MRI was normal. He had Refractory epilepsy with multiple drug resistance which was finally controlled with epidiolex (Cannabidiol). | An additional likely pathogenic heterozygous variant c.3151G>T (p.Gly1051Trp) in POLG gene was identified as related with autosomal recessive epilepsy conditions, inherited from the father. An additional three VUSs were identified: c.155T>C (p.Phe52Ser) in AMT, c.737T>C (p.Met246Thr) in MECP2 and c.2941A>T (p.Ser981Cys) in RELN. The genetic combination and the variant identified in MECP2 were detected in the mother, and the VUSs identified in the RELN and AMT were inherited from the father as well. |
| Case 3 | c.7790T>C (p.Phe2597Ser and c.7805C>T (p.Thr2602Met) in RANBP2 and c.1703A>G (p.Glu568Gly) in RYR3 were identified, each inherited from another parent. Manifested with Tinnitus suspected to be temporal auditory epilepsy. He has Tourette syndrome and ADHD. MRI was normal. His symptoms concluded to be non epilpeptic and he wasn't treated with antiepileptic medications | He had another one VUS in the epilepsy panel c.2099T>C (p.Ile700Thr) in the DIAPH gene inherited from the mother. |
